# Supplementary figures and images for: KAT6A is essential for developmental control gene expression in neural stem and progenitor cells
Source: PLoS Genet. 2026 May 4;22(5):e1012133. doi: 10.1371/journal.pgen.1012133 (PMC13160438; doi:10.1371/journal.pgen.1012133)

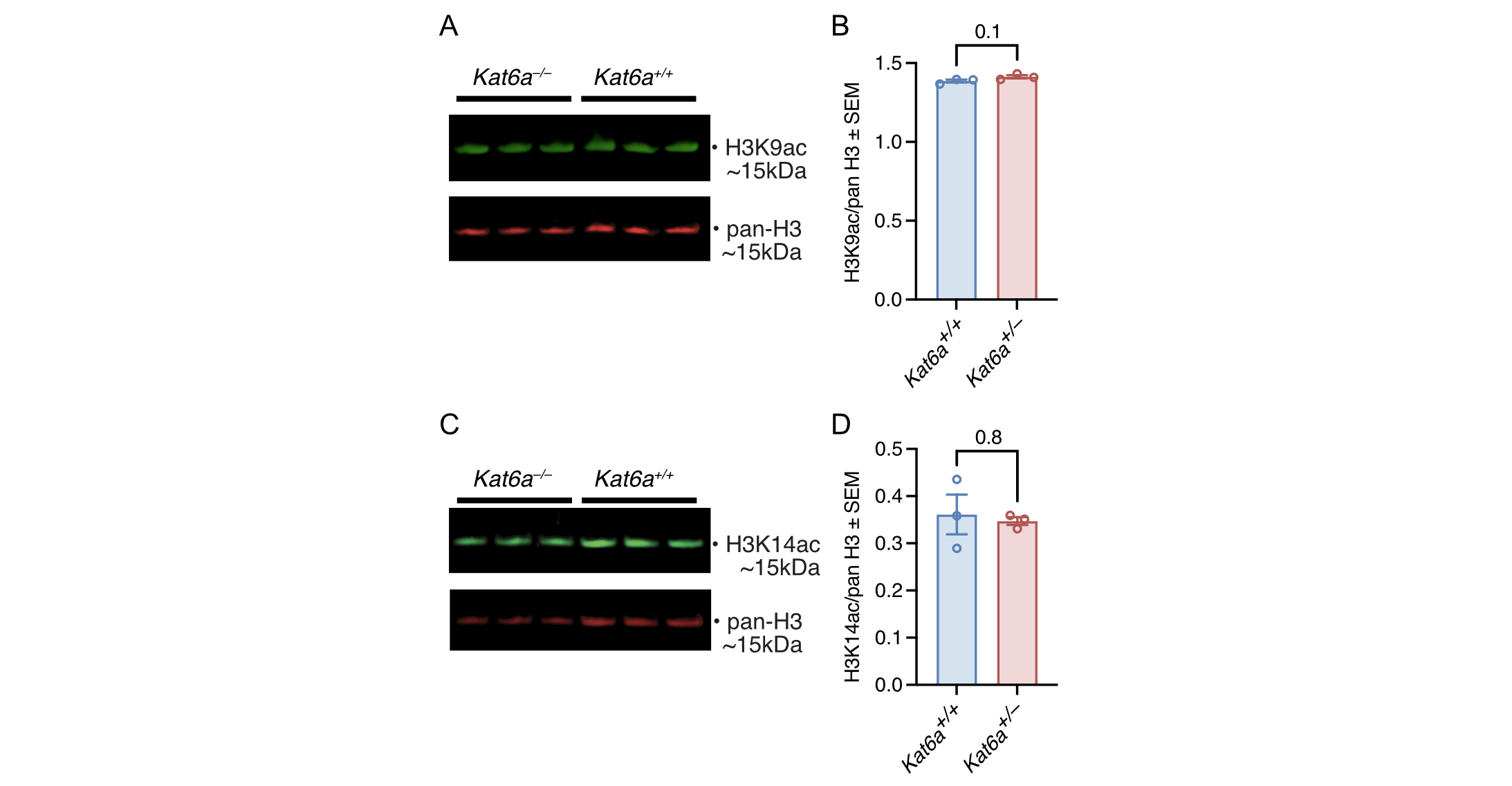

Supplement: S1 Fig — Loss of KAT6A does not affect global H3K9ac or H3K14ac levels in proliferating neural stem and progenitor cells (NSPCs). (A,B) H3K9 acetylation levels and pan histone H3 levels assessed by Western blotting (A) and densitometry (B) in Kat6a+/+ and Kat6a–/– NSPCs. Each lane was loaded with 0.5 µg of acid extracted protein from NSPCs isolated from an individual mouse embryo. H3K9ac levels were normalised to pan-H3 levels. (C,D) H3K14 acetylation levels and pan histone H3 levels assessed by Western blotting (C) and densitometry (D) in Kat6a+/+ and Kat6a–/– NSPCs. Each lane was loaded with 2 µg of acid extracted protein from NSPCs isolated from an individual mouse embryo. H3K14ac levels were normalised to pan-H3 levels. NSPC isolates from N = 3 E12.5 embryos per genotype. Each circle in (B,D) represents NSPCs isolated from an individual mouse embryo. Data are presented as mean ± SEM and were analysed by unpaired, two-tailed Student’s t-test (B,D). (TIFF) [file pgen.1012133.s001.tiff]

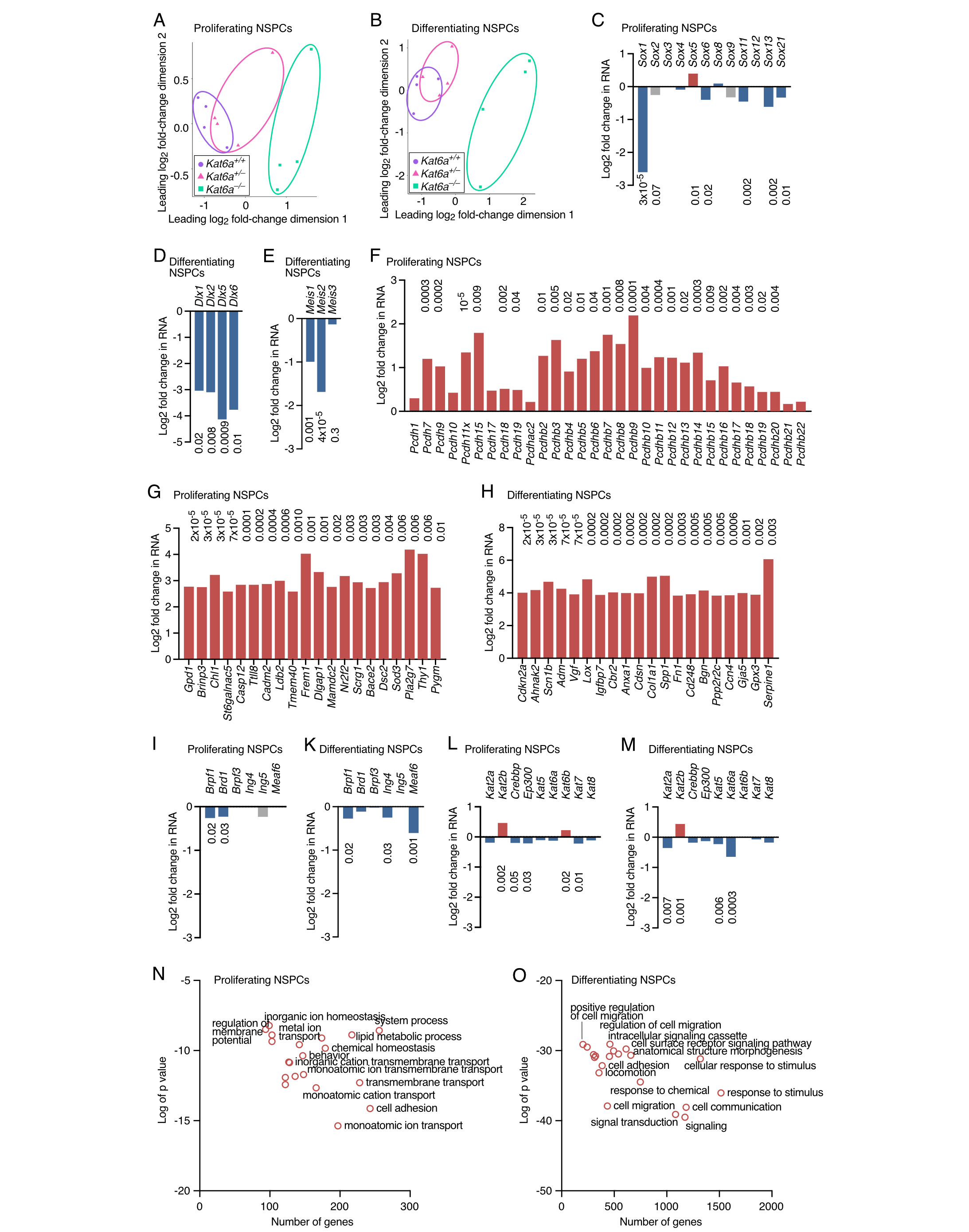

Supplement: S2 Fig — Loss of KAT6A affects gene expression in proliferating and differentiating neural stem and progenitor cells (NSPCs). (A-O) RNA sequencing data of NSPCs isolated from N = 4 Kat6a+/+, 4 Kat6a+/– and 4 Kat6a–/– E12.5 embryos. Data were analysed as described in the methods section under RNA sequencing data analysis. Differences in gene expression with a false discovery rate (FDR) < 0.05 were considered significant. (A,B) Multidimensional scaling plot of the leading gene expression differences between samples in pair-wise comparisons of proliferating (A) and differentiating (B) Kat6a+/+, Kat6a+/– and Kat6a–/– NSPC samples. (C) Log2 fold-change in RNA levels of SOX genes in proliferating Kat6a–/– vs. Kat6a+/+ NSPCs. FDRs shown below the bars. Genes that are downregulated or upregulated with transcriptome-wide significance are indicated with blue and red bars, respectively. Gene not changed are indicated in grey bars. (D,E) Log2 fold-change in RNA levels of DLX genes (D) and MEIS genes (E) in differentiating Kat6a–/– and Kat6a+/+ NSPCs. FDRs shown below the bars. (F) Log2 fold-change in RNA levels of protocadherin genes in proliferating Kat6a–/– vs. Kat6a+/+ NSPCs. FDRs shown above the bars. (G,H) Log2 fold-change in RNA levels of the top 20 genes (by fold-change amplitude, average expression > 1 CPM) upregulated in proliferating (G) and differentiating (H) Kat6a–/– vs. Kat6a+/+ NSPCs. FDRs shown above the bars. (I,K) Log2 fold-change in RNA levels of genes encoding components of the KAT6A protein complex in proliferating (I) and differentiating (K) Kat6a–/– vs. Kat6a+/+ NSPCs. FDRs shown below the bars. (L,M) Log2 fold-change in RNA levels of histone acetyltransferase genes in proliferating (L) and differentiating (M) Kat6a–/– vs. Kat6a+/+ NSPCs. FDRs shown below the bars. (N,O) Top 20 gene ontology terms (biological process) associated with genes upregulated in proliferating (N) and differentiating (O) Kat6a–/– vs. Kat6a+/+ NSPCs. (TIFF) [file pgen.1012133.s002.tiff]

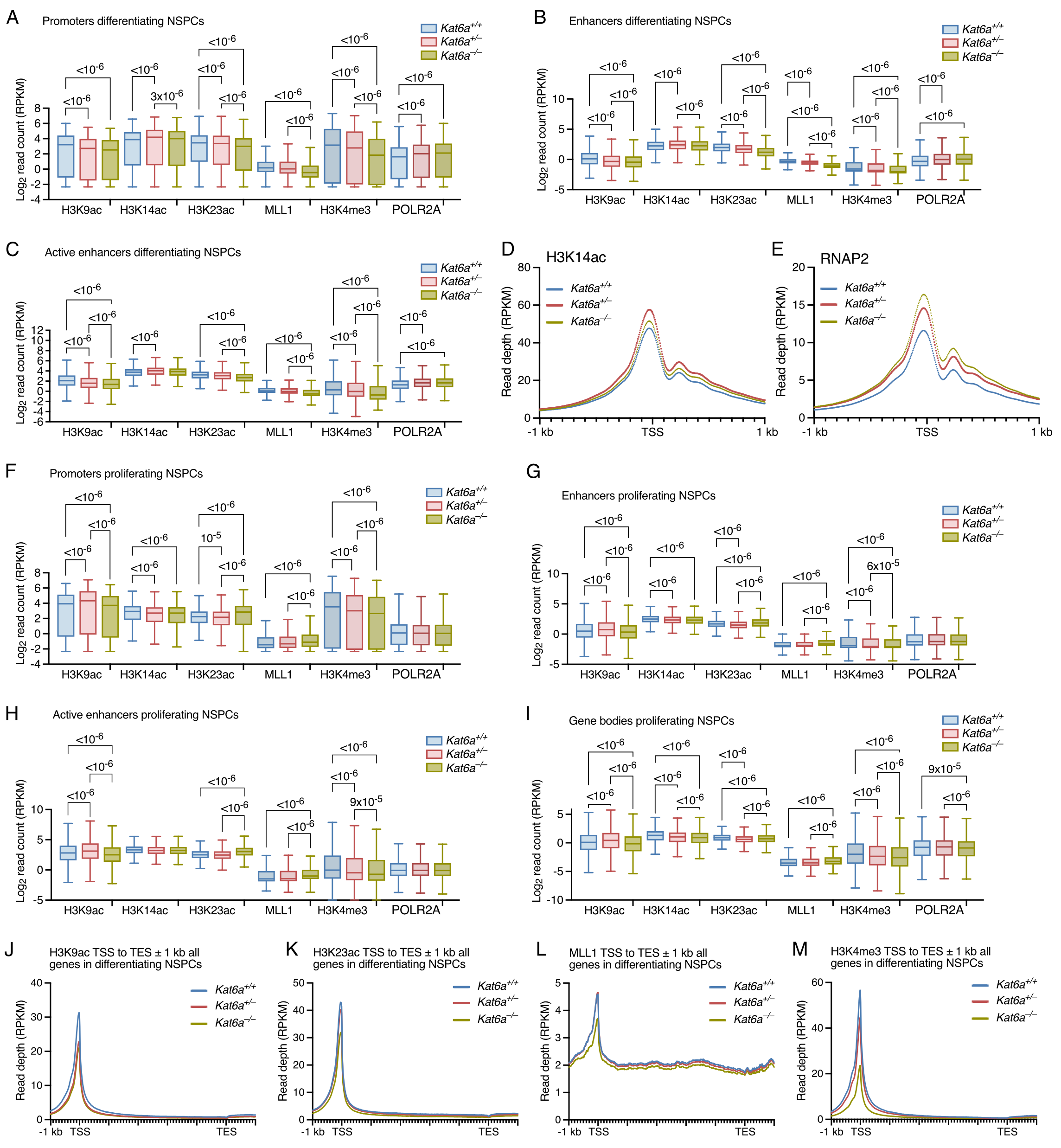

Supplement: S3 Fig — Loss of KAT6A causes broad changes in H3K9ac, H3K23ac and H3K4me3 levels as well as MLL1 occupancy that are more pronounced in differentiating than in proliferating NSPCs. (A-I) CUT&Tag results of NSPCs isolated from N = 3 Kat6a+/+, 4 Kat6a+/– and 3 Kat6a–/– E12.5 embryos. Data were analysed as described in the methods section under Automated CUT&Tag sequencing data analysis. Differences in occupancy with a false discovery rate (FDR) < 0.05 were considered significant. Data in (A-C,F-I) were analysed by Kruskal-Wallis test followed by Dunn’s correction for multiple testing. (A-C) Log2 of CUT&Tag read count per kilobase normalised to library size (RPKM) and genomic feature length, accrued over promoters (A), enhancers (B) and active enhancers (C) in differentiating Kat6a+/+, Kat6a+/– and Kat6a–/– NSPCs detecting H3K9ac, H3K14ac, H3K23ac, MLL1, H3K4me3 and POLR2A. (D,E) Read depth aggregates over all protein coding genes for H3K14ac (D) and POLR2A (E) over the interval from -1 kb to +1 kb of the transcription start site (TSS) in differentiating Kat6a+/+, Kat6a+/– and Kat6a–/– NSPCs. (F-I) Log2 of CUT&Tag read count per kilobase normalised to library size (RPKM) and genomic feature length, accrued over promoters (F), enhancers (G), active enhancers (H) and gene bodies (I) in proliferating Kat6a+/+, Kat6a+/– and Kat6a–/– NSPCs detecting H3K9ac, H3K14ac, H3K23ac, MLL1, H3K4me3 and POLR2A. (J-M) Read depth aggregates over all protein coding genes for H3K9ac (J), H3K23ac (K), MLL1 (L) and H3K4me3 (M) over the interval from -1 kb upstream of the transcription start site (TSS) to +1 kb downstream of the transcription end site (TES) in differentiating Kat6a+/+, Kat6a+/– and Kat6a–/– NSPCs. Enhancers were defined as H3K4me1 enriched regions (GSM2406793) outside of promoters; active enhancers as H3K27ac (GSM2406793; [99]) and H3K4me1 (GSM2406791; [99]) enriched regions outside of promoters. (TIFF) [file pgen.1012133.s003.tiff]

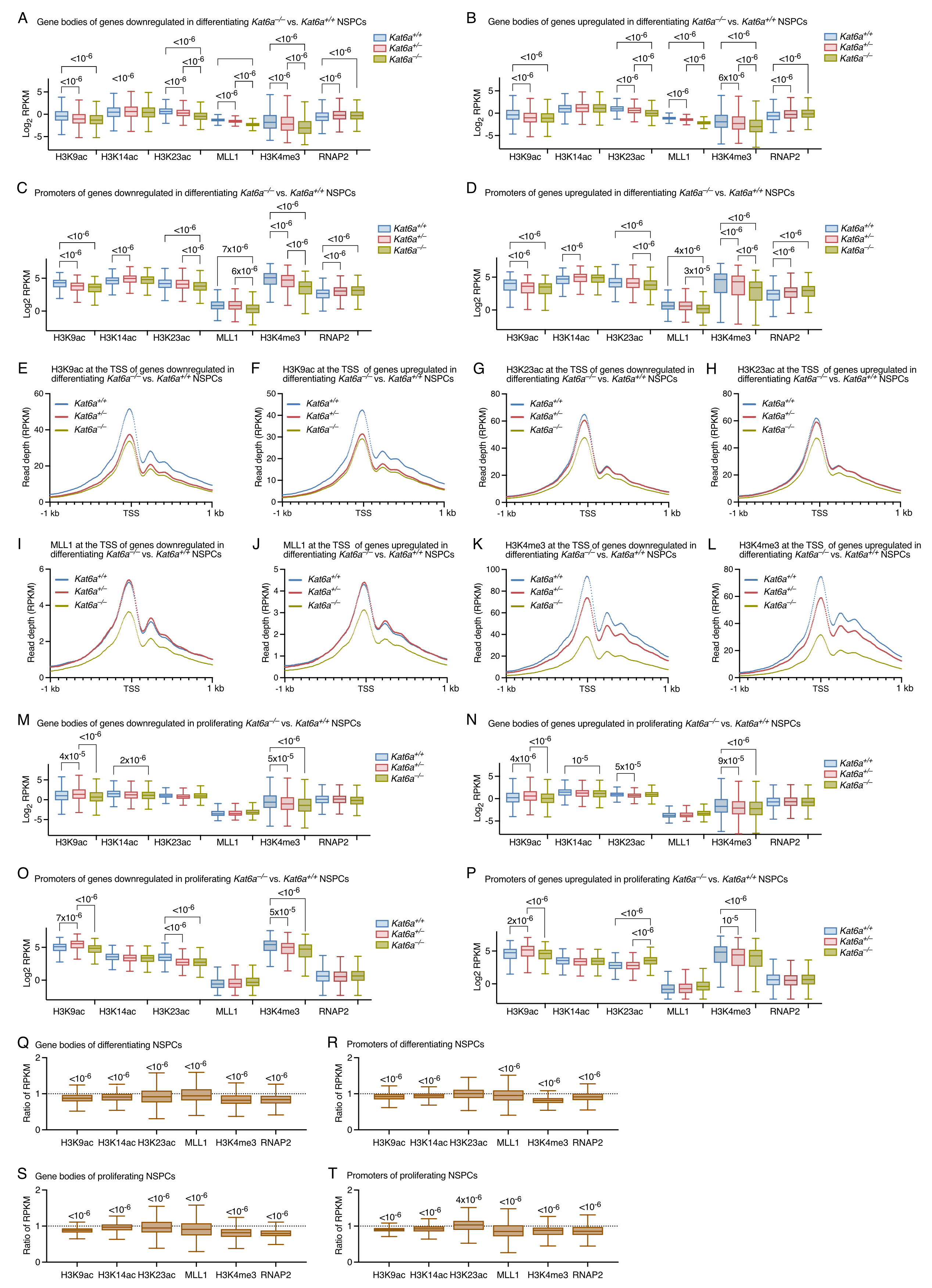

Supplement: S4 Fig — The changes in H3K9ac, H3K23ac, H3K4me3 levels and MLL1 occupancy caused by loss of KAT6A are only modestly more pronounced in genes that are downregulated than in genes that are upregulated in differentiating and proliferating NSPCs. (A-T) CUT&Tag results of NSPCs isolated from N = 3 Kat6a+/+, 4 Kat6a+/– and 3 Kat6a–/– E12.5 embryos. Data were analysed as described in the methods section under Automated CUT&Tag sequencing data analysis. Differences in occupancy with a false discovery rate (FDR) < 0.05 were considered significant. Data in (A-D,M-P) were analysed by Kruskal-Wallis test followed by Dunn’s correction for multiple testing. Data in (Q-T) are shown as Tukey box and whisker plots and were analysed by one sample Wilcoxon test compared to a theoretical value of 1. (A-D) Log2 of CUT&Tag read count per kilobase normalised to library size (RPKM) and genomic feature length, accrued over gene bodies (A,B) and promoters (C,D) of genes that are downregulated (A,C) and upregulated (D,E) based on RNA-seq results in differentiating Kat6a–/– vs. Kat6a+/+NSPCs. (E-L) Read depth aggregates over protein coding genes that are downregulated (E,G,I,K) and upregulated (F,H,J,L) in differentiating Kat6a+/+, Kat6a+/– and Kat6a–/– NSPCs for H3K9ac (E,F), H3K23ac (G,H), MLL1 (I,J) and H3K4me3 (K,L) over the interval from -1 kb to +1 kb of the transcription start site (TSS) in differentiating Kat6a–/– vs. Kat6a+/+ NSPCs. (M-P) Log2 of CUT&Tag read count per kilobase normalised to library size (RPKM) and genomic feature length, accrued over gene bodies (M,N) and promoters (O,P) of genes that are downregulated (M,O) and upregulated (N,P) in proliferating Kat6a+/+, Kat6a+/– and Kat6a–/– NSPCs. (Q-T) CUT&Tag read counts ratios in genes downregulated to upregulated in Kat6a–/– vs. Kat6a+/+ differentiating (Q,R) and proliferating (S,T) NSPCs (normalised to upregulated genes) in the gene bodies (Q,S) and promoters (R,T) of genes. (TIFF) [file pgen.1012133.s004.tiff]

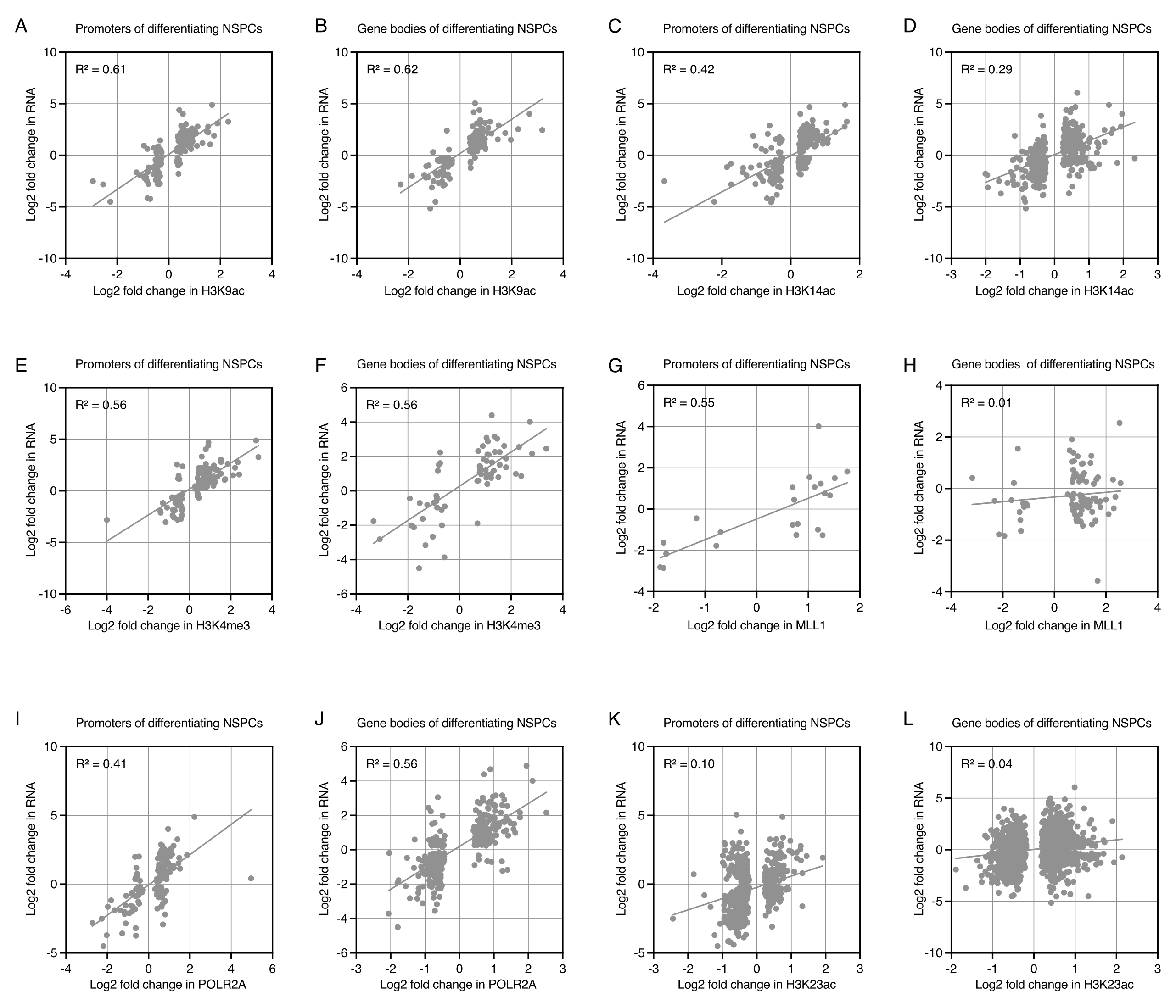

Supplement: S5 Fig — Histone acetylation levels and POLR2A occupancy changes compared to changes in RNA levels between differentiating Kat6a+/+ and Kat6a–/– NSPCs. (A-L) RNA sequencing and CUT&Tag sequencing results of differentiating NSPCs isolated from N = 3–4 Kat6a+/+ and 3–4 Kat6a–/– E12.5 embryos. Data were analysed as described in the methods section under RNA sequencing data analysis and Automated CUT&Tag sequencing data analysis. Differences in gene expression or occupancy with a false discovery rate (FDR) < 0.05 were considered significant. (A-L) Correlation between log2 fold-change in RNA levels and log2 fold-change in H3K9ac (A,B), H3K14ac (C,D), H3K4me3 levels (E,F), MLL1 (G,H), POLR2A occupancy (I,J) and H3K23ac (K,L) in differentiating Kat6a–/– vs. Kat6a+/+ NSPCs at promoters (A,C,E,G,I,K) and bodies (B,D,F,H,J,L) of protein coding genes differentially expressed (FDR < 0.05) and differentially occupied (FDR < 0.05), except for MLL1, which displayed too few differences at FDR < 0.05 to allow a simple linear regression, thus p < 0.001 for promoters and p < 0.01 for gene bodies were used to give an indication of the relationship of MLL1 with RNA levels in differentiating NSPCs). (TIFF) [file pgen.1012133.s005.tiff]

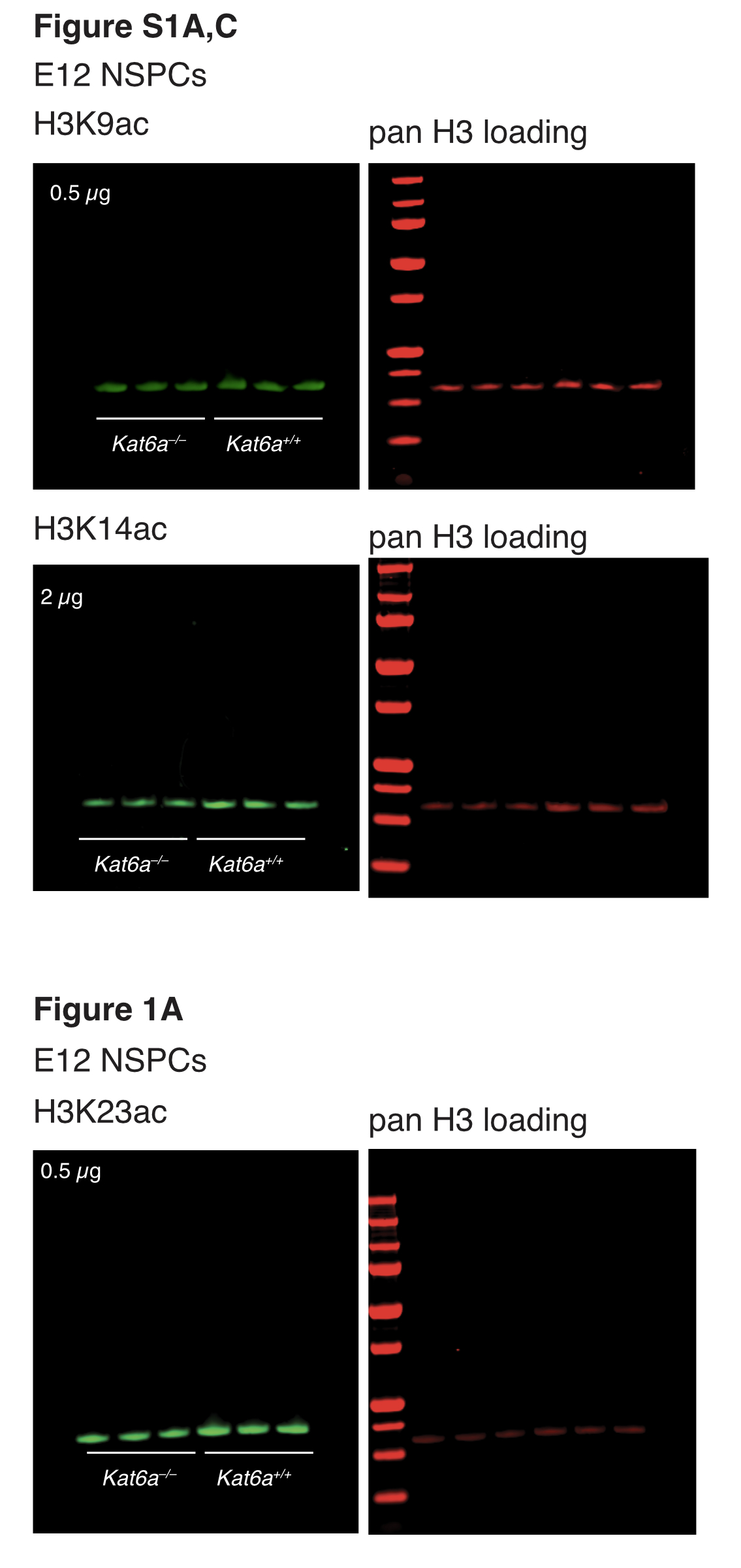

Supplement: S1 Raw Gels — This file contains the uncropped images of western blots displayed in Figs 1A, S1A and S1B. (JPG) [file pgen.1012133.s006.jpg]
