# Supplementary material for: KAT6A is essential for developmental control gene expression in neural stem and progenitor cells
Source: PLoS Genet. 2026 May 4;22(5):e1012133. doi: 10.1371/journal.pgen.1012133 (PMC13160438; doi:10.1371/journal.pgen.1012133)
Supplement: Attachment — Submitted filename: Responses to Reviewers 27032026v5.docx [file pgen.1012133.s015.docx]

PGENETICS-D-26-00057

**Point-by-point responses to the reviewer**

We would like to thank the editors and the reviewers for the time and effort spent on evaluating our manuscript and thank them for their insightful comments. We believe that our changes in response to the reviewers’ comments have improved the manuscript and hope that it is now acceptable for publication in PLoS Genetics.

**Reviewers' comments with author responses inserted**

**Reviewer #1**

**Reviewer #1:** Voss et al. characterized the Kat6a knockout mouse with a focus on the properties of neural stem and progenitor cells (NSPCs) in in vitro differentiation settings. Homozygous loss of Kat6a resulted in slower proliferation and weaker expression of a differentiation marker in NSPCs. RNA-seq analyses revealed a set of genes affected by the loss of Kat6a. They also analyzed the epigenomic landscape of NSPCs by CUT&Tag chromatin profiling and showed that the presence of H3K9ac, H3K23ac, MLL1, and H3K4me3 was reduced by loss of Kat6a in differentiating NSPCs. By comparing the expression profile data and the epigenetic landscape data, they showed that the presence of MLL1 and H3K4me3 correlates with gene expression changes induced by Kat6a loss.

Information on the primary culture cells from the KO mice is always important and appreciated by the field. I always appreciate this group’s effort of their detailed analyses on genetically engineered animals. However, I felt the conclusions regarding what causes what are vague and the manuscript can be improved by analyzing differently.

**Response:** We thank the reviewer the positive comments regarding our work and for pointing out the importance studying primary KO vs. WT cells, as well as the suggestion to analyse the data in a different way, which we have now incorporated in the revised version of the manuscript.

**Reviewer #1:** Followings are my suggestions:
Major points:
Kat6a-/- NSPCs proliferate slowly and express a subset of genes lowly and another subset of genes highly compared to the wildtype. Yet the epigenetic landscapes were analyzed as a whole in Figure 3. I would be more curious about what’s going on in the genes down-regulated by loss of Kat6a. I recommend the authors to do the analyses like Figure 3 on the gene sets of down-regulated and up-regulated separately.

**Response:** As the reviewer suggested, we have now performed the same analyses (as in Figure 3) separately on the genes that were downregulated and genes that were upregulated separately. The new data are displayed in a new Figure S4.

**Reviewer #1:** I wonder how homogeneous the NSPC pools are especially in the differentiating conditions. The observed changes among the genotypes may be due to different demographics of the NSPCs. Single cell RNA-seq analysis might help.

**Response:** We agree with the reviewer that neural stem and progenitor cells (NSPCs) are not a homogeneous cell population, particularly in the differentiating condition. Our marker gene analysis suggests that close to 100% of the proliferating NSPCs express the neural stem and progenitor cell protein SOX2 (Figure 1D). Nevertheless, there could be a different composition of stem cells vs. progenitor cells between *Kat6a* genotypes. Under differentiation conditions, we found a gene-dose dependent reduction in ßIII-tubulin positive neurons in the absence of one and two alleles of *Kat6a*, indicating that the composition of differentiating NSPCs is clearly different between genotypes. As the reviewer suggested, single-cell RNA-seq might provide further insights. However, Drs Thomas and Voss are now retired, and therefore do not have the opportunity to respond to this request with further experiments. To address this very valid comment, we have included a discussion of this caveat in a new section of the discussion entitled *“Limitations of the study”* on pages 19 and 20, also including the suggestion that single-cell RNA-seq might provide further insights. We sincerely hope that the reviewer is satisfied with this revision.

**Reviewer #1:**

Minor points:
On page 10, “provides an inside into major changes” may be a typo.
“insight” instead of “inside”?

**Response:** We thank the reviewer for picking up this mistake and have corrected it.

**Reviewer #1:** In Figure 3B-E, localizations of various histone modifications and MLL1 are shown. As a comparison, the same analysis on the factors that behave similarly among the genotypes should be shown (Pol II, H3K14ac?)

**Response:** As the reviewer suggested, we have now included the TSS coverage plots that show no clear genotype effect in supplemental Figure S3D and S3E.

**Reviewer #2**

**Reviewer #2:** Review of PGENETICS-D-26-00057:
In this manuscript, Voss et al reports the important role of mouse lysine acetyltransferase 6A (KAT6A) in regulating gene expression in neural stem and progenitor cells. For this, such cells were cultured from wild-type, heterozygous and homozygous knockout embryos at E12.5 due to lethality of homozygous knockout embryos at E13.5. Impact of Kat6a loss on histone acetylation, cell proliferation/differentiation, gene expression (via RNA-Seq) and genome-wide distribution of several histone acetylation marks (by CUT&TAG) was assessed. The results identify important roles of KAT6A in regulating the expression of genes essential for neural stem and progenitor cells, which is consistent with what is known in the literature. This study also complements nicely elegant works that have been published about KAT6A and its paralog, KAT6B, by the two groups led by Voss and Thomas, and should thus be of interests to many others working on epigenetic and development. The following issues are suggested for the authors to consider when they revise the manuscript for formal publication.

**Response:** We would like to thank the reviewer for the positive comments.

**Reviewer #2:** 1) On page 2 (top), the statements on BRPF1, 2 and 3 are inaccurate and do not reflect the current state of knowledge, as it is typically considered by many in the field that BRPF2 and BRPF3 act through KAT7, instead KAT6A/B. The related literature should be discussed.

**Response:** We agree with the reviewer that the available evidence suggest that the protein complex containing either KAT6A or KAT6B as the lysine acetyltransferase most likely contains BRPF1 rather than BRPF2 or 3. Our sentence on page two was referring to the first paper identifying these protein complexes (Doyon et al., Mol Cell 2006), where all three BRPFs, as well as KAT6A (MOZ), KAT6B (MORF) and KAT7 were pulled down with ING5. We agree that since then more details have been published and we have now incorporated these into the revised version of our paper on page 4, as the reviewer suggested.

**Reviewer #2:** 2) It is well established that BRPF1 serves as a key regulator of KAT6A/B, and its role in neural stem/progenitor cells has been well established. This issue can be discussed in the manuscript.

**Response:** As the reviewer suggested, we have now included a discussion of the role of BRPF1 in neural stem and progenitor cells on page 7 of our revised manuscript.

**Reviewer #2:** 3) Because the RNA-seq data underpin many of the conclusions, independent validation (qRT–PCR of representative Sox, Dlx, Meis, or Hox targets) could be included.

**Response:** We agree with the reviewer that it would be desirable to confirm some of our RNA-seq results, for example by RT-qPCR. However, Drs Thomas and Voss are now retired, and therefore do not have the opportunity to respond to this request with further experiments. To address the reviewer's comment, we have now pointed out the need to confirm the RNA-seq results in a new section entitled *“Limitations of the study”* pages 19 and 20. We very much hope that the reviewer will be satisfied with this addition to the text.

**Reviewer #2:** 4) The proposed functional interplay between KAT6A and MLL1 remains largely correlative. Experiments showing a physical or functional interaction would significantly strengthen the model and help clarify whether KAT6A acts through catalytic activity, structural roles, or both.

**Response:** We agree with the reviewer that it would be really interesting to explore the functional relationship between KAT6A and MLL1 in much more detail. In fact, this is one of the research questions that we really regret not being able to pursue anymore, since we are retired. To address the reviewers comment we have included an extensive discussion of this point in a new section of the discussion entitled *“Limitations of the study”* on pages 19 and 20*.* In addition, as we now discuss on pages 16, 17 and 19, a paper reporting that endogenous KAT6A (MOZ) and the endogenous C-terminal fragment of MLL1 were co-immunoprecipitated from HEK293T and K562 cell lysates (Paggetti et al., Oncogene 2010). Perhaps we should have cited this study in our original version of the paper; however, we note that Paggetti and colleagues focused on the hematopoietic system rather than neural stem cell proliferation and differentiation. We hope that the reviewer will be satisfied with this addition to the text.

**Reviewer #2:** 5) While H3K23ac is presented as a central enzymatic output, its changes do not consistently align with the transcriptional effects described.

**Response:** Yes, we agree and had stated this in the original version of our manuscript on page 12, now page 13 and discussed this phenomenon on pages 13 and 14, now pages 14 and 15.

**Reviewer #2:** 6) The manuscript is well written, but it is always wise to double-check its text for accuracy and readability.

**Response:** As the reviewer requested, we have read and re-read the manuscript to check the manuscript for accuracy and readability again.

**Reviewer #2:** 7) RNA-Seq and CUT&TAG data should be deposited in public databases, and the accession numbers should be included in the revised manuscript.

**Response:** As stated in our original manuscript, the RNA-seq and CUT&Tag data have already been deposited in the GEO database and are accessible to reviewers using GEO accession number: GSE314079; Reviewer token: qzopmouabjybpsj and GEO accession number: GSE311724; Reviewer token: cfktuacoflyxxwh, respectively. The data will be made publicly available upon acceptance of the paper. We have checked these accession numbers and reviewer tokens again and they worked. Please use this link to search for the data: https://www.ncbi.nlm.nih.gov/geo/query/acc.cgi

**Reviewer #3**

**Reviewer #3:** This study provides new insights into the role of KAT6A in neural stem and progenitor cells by integrating proliferation, differentiation, transcriptomic, and chromatin profiling data. Beyond confirming a proliferation defect, the authors demonstrate impaired neuronal differentiation and widespread transcriptional dysregulation upon Kat6a loss, accompanied by a reproducible global ~50% reduction in H3K23ac and gene-specific reductions in H3K9ac, H3K14ac, H3K4me3, MLL1 occupancy, and POLR2A. Unexpectedly, reduced KAT6A mediated H3K23ac did not correlate with expression changes, contrasted with the strong positive correlation between altered MLL1 occupancy/H3K4me3 and transcription. Therefore, the authors suggest a two-function model in which KAT6A acts both as a global H3K23 acetyltransferase maintaining chromatin accessibility and as a facilitator of MLL1 recruitment at developmental regulators such as HOX, DLX, TBX, and SOX genes (also perhaps competence for the binding of repressor proteins?). Overall, there are no major criticisms of the study as the data is of high quality. Nevertheless, the authors need to address in the discussion the limitations of their study and proposed model, and future experiments that could address these limitations. For example, how to establish a direct physical or mechanistic interaction between KAT6A and MLL1 to determine whether the observed correlation reflects causation, indirect chromatin effects, or secondary consequences of altered transcription. How to explore the basis for the global reduction, but not the complete loss of H3K23ac and the identity of compensatory acetyltransferase(s). In other words, how to functional validate the proposed two-function model.

**Response:** We thank you reviewer for the positive comments made about the quality of our data. We fully agree with the limitations mentioned by the reviewer. To thoroughly address the reviewer’s comment, we have now created a new section of the discussion entitled *“Limitation of the study”* on pages 19 and 20, where we now address these discussion points to make the caveats of the study clear.

Relating to one of the caveats, we now discuss on pages 16, 17 and 19 a paper reporting that endogenous KAT6A (MOZ) and the endogenous C-terminal fragment of MLL1 were co-immunoprecipitated from HEK293T and K562 cell lysates (Paggetti et al., Oncogene 2010). Perhaps we should have cited this study in our original version of the paper; however, we note that Paggetti and colleagues focused on the hematopoietic system rather than neural stem cell proliferation and differentiation.

**Reviewer #3:** Other questions include :

1) E12.5 was chosen. Would an earlier time point have been more informative i.e. to observe the primary changes in gene expression and not secondary affects given that the embryos die shortly after?

**Response:** At E12.5 the *Kat6a^–/–^* embryos are healthy and normal. *Kat6a^–/–^* foetuses die between E13.5 and E14.5 on the C57BL/6 genetic background that we used. While this may sound soon, mouse gestation proceeds very rapidly. It only takes 19.5 days in total. Mouse embryos display very obvious progress in development and size within even 12 h. Therefore, 24 h is quite removed from the time of death. Furthermore, *Kat6a^–/–^* foetuses do not die of a defect in the forebrain, which is the source of our neural stem and progenitor cells, but rather die of a cardiovascular defect, which does not manifest any signs (e.g. oedema) before E13.5/E14.0, indicating that heart and large vessel function is adequate at E12.5.

**Reviewer #3:** 2) Regarding Figure 3A, it would be informative to show metagene plots from upstream of the TSS to down stream of the transcription termination site;

**Response:** As the reviewer suggested, we have generated metagene plots from TSS-1kb to TES+1kb. The new results are displayed in Figure S3J to S3M.

**Reviewer #3:** 3) How do you explain the increase in Pol II in the gene body in KAT6A knockout cells (Fig. 3A);

**Response:** We can only speculate at this point that Pol II might progress through gene loci more slowly in the *Kat6a^–/–^* NSPCs compared to the *Kat6a^+/+^* cells. To address the reviewer’s comment, we have now stated this possibility on page 10.

**Reviewer #3:** 4) For the different H3 acetylation sites, is the temporal order for their establishment known? Which acetylation sites precede transcription, and which are a consequence of transcription?

**Response:** The temporal order of histone acetylation is not known. The turnover of histone acetylation is known to range from less than 10 minutes to 8 hours (Weinert et al., Cell 2016). The causal relationship between histone acetylation and gene transcription has long been debated and a resolution of this question has only recently emerged. Peak histone acetylation at some histone lysine residues temporally precedes peak mRNA production in yeast (Kuang et al., Nat Struct Mol Biol 2014); histone acetylation facilitates de novo initiation of gene expression in mouse NSPCs (Kueh et al., Cell Reports 2023); and three ways of chemical inhibition of transcription has no effects on histone acetylation levels in mammalian cells (Liebner et al., Nat Comms 2024), whereas chemical inhibition of histone lysine acetyltransferases affects gene expression (Baell et al, Nature 2018; Sharma et al., Cell Chem Biol 2023; Liebner et al., Nat Comms 2024). To address the reviewer’s comment, we now discuss the causal relationship on page 20.

**Have all data underlying the figures and results presented in the manuscript been provided?**
Large-scale datasets should be made available via a public repository as described in the *PLOS Genetics* [data availability policy](https://urldefense.com/v3/__http:/track.editorialmanager.com/CL0/http:*2F*2Fjournals.plos.org*2Fplosgenetics*2Fs*2Fdata-availability/1/010f019c7d02f271-cb24c58a-b873-43d4-8ce6-8743bea9e2cd-000000/dAabDKWHvCUMnmKbfZ71H_s3mb65MoVAOiVO2e5djkA=251__;JSUlJSU!!KKt0acMt!fvLT9e-6YqotZdmorkmgeSWxa9zGR4u1sfstnxvzrtSCK--0ngE_hpcqGpgQX1j_6b284vK3qatDYurr$), and numerical data that underlies graphs or summary statistics should be provided in spreadsheet form as supporting information.

Reviewer #1: Yes

Reviewer #2: Yes

Reviewer #3: Yes

PLOS authors have the option to publish the peer review history of their article ([what does this mean?](https://urldefense.com/v3/__https:/track.editorialmanager.com/CL0/https:*2F*2Fjournals.plos.org*2Fplosgenetics*2Fs*2Feditorial-and-peer-review-process*23loc-peer-review-history/1/010f019c7d02f271-cb24c58a-b873-43d4-8ce6-8743bea9e2cd-000000/QqQcsUPadxvXAZI0mJclRlykkTpU3CE4E2EsIHDkCGE=251__;JSUlJSUl!!KKt0acMt!fvLT9e-6YqotZdmorkmgeSWxa9zGR4u1sfstnxvzrtSCK--0ngE_hpcqGpgQX1j_6b284vK3qVawnH2s$)). If published, this will include your full peer review and any attached files.


If you choose “no”, your identity will remain anonymous but your review may still be made public.


**Do you want your identity to be public for this peer review?** For information about this choice, including consent withdrawal, please see our [Privacy Policy](https://urldefense.com/v3/__https:/track.editorialmanager.com/CL0/https:*2F*2Fwww.plos.org*2Fprivacy-policy/1/010f019c7d02f271-cb24c58a-b873-43d4-8ce6-8743bea9e2cd-000000/1YtraW07HJKge-oLpDZUwzunixztVvOR6_vr_fUpdag=251__;JSUl!!KKt0acMt!fvLT9e-6YqotZdmorkmgeSWxa9zGR4u1sfstnxvzrtSCK--0ngE_hpcqGpgQX1j_6b284vK3qVVOJb2H$).

Reviewer #1: No

Reviewer #2: No

Reviewer #3: No

[NOTE: If reviewer comments were submitted as an attachment file, they will be attached to this email and accessible via the submission site. Please log into your account, locate the manuscript record, and check for the action link "View Attachments". If this link does not appear, there are no attachment files.]

**Figure resubmission:**

While revising your submission, we strongly recommend that you use PLOS’s NAAS tool ([https://ngplosjournals.pagemajik.ai/artanalysis](https://urldefense.com/v3/__https:/ngplosjournals.pagemajik.ai/artanalysis__;!!KKt0acMt!fvLT9e-6YqotZdmorkmgeSWxa9zGR4u1sfstnxvzrtSCK--0ngE_hpcqGpgQX1j_6b284vK3qU6Y64la$)) to test your figure files. NAAS can convert your figure files to the TIFF file type and meet basic requirements (such as print size, resolution), or provide you with a report on issues that do not meet our requirements and that NAAS cannot fix.

After uploading your figures to PLOS’s NAAS tool - [https://ngplosjournals.pagemajik.ai/artanalysis](https://urldefense.com/v3/__https:/ngplosjournals.pagemajik.ai/artanalysis__;!!KKt0acMt!fvLT9e-6YqotZdmorkmgeSWxa9zGR4u1sfstnxvzrtSCK--0ngE_hpcqGpgQX1j_6b284vK3qU6Y64la$), NAAS will process the files provided and display the results in the "Uploaded Files" section of the page as the processing is complete. If the uploaded figures meet our requirements (or NAAS is able to fix the files to meet our requirements), the figure will be marked as "fixed" above. If NAAS is unable to fix the files, a red "failed" label will appear above. When NAAS has confirmed that the figure files meet our requirements, please download the file via the download option, and include these NAAS processed figure files when submitting your revised manuscript.

**Reproducibility:**

To enhance the reproducibility of your results, we recommend that authors deposit laboratory protocols in protocols.io, where a protocol can be assigned its own identifier (DOI) such that it can be cited independently in the future. Additionally, PLOS ONE offers an option to publish peer-reviewed clinical study protocols. Read more information on sharing protocols at [https://plos.org/protocols?utm_medium=editorial-email&utm_source=authorletters&utm_campaign=protocols](https://urldefense.com/v3/__https:/plos.org/protocols?utm_medium=editorial-email&utm_source=authorletters&utm_campaign=protocols__;!!KKt0acMt!fvLT9e-6YqotZdmorkmgeSWxa9zGR4u1sfstnxvzrtSCK--0ngE_hpcqGpgQX1j_6b284vK3qQtZs3z2$)

*In compliance with data protection regulations, you may request that we remove your personal registration details at any time.* [*(Remove my information/details)*](https://urldefense.com/v3/__https:/track.editorialmanager.com/CL0/https:*2F*2Fwww.editorialmanager.com*2Fpgenetics*2Flogin.asp*3Fa=r/1/010f019c7d02f271-cb24c58a-b873-43d4-8ce6-8743bea9e2cd-000000/3hNzetjAfsdZnLSA4g-2IObpyuwhq51IAw_T4JdvEDo=251__;JSUlJSU!!KKt0acMt!fvLT9e-6YqotZdmorkmgeSWxa9zGR4u1sfstnxvzrtSCK--0ngE_hpcqGpgQX1j_6b284vK3qZaEjlU5$)*. Please contact the publication office if you have any questions.*
